# Effects of mandibular setback with or without maxillary advancement osteotomies on pharyngeal airways: An overview of systematic reviews

**DOI:** 10.1371/journal.pone.0185951

**Published:** 2017-10-09

**Authors:** Su Keng Tan, Wai Keung Leung, Alexander Tin Hong Tang, Roger A. Zwahlen

**Affiliations:** 1 Center of Oral & Maxillofacial Surgery Studies, Faculty of Dentistry, Universiti Teknologi MARA Sungai Buloh Campus, Jalan Hospital, Selangor Darul Ehsan, Malaysia; 2 Discipline of Oral and Maxillofacial Surgery, Faculty of Dentistry, University of Hong Kong, Hong Kong SAR, PR China; 3 Discipline of Periodontology, Faculty of Dentistry, The University of Hong Kong, Prince Philip Dental Hospital, Sai Ying Pun, Hong Kong SAR, PR China; 4 Orthodontist in private practice, 503 Tak Shing House, Central, Hong Kong SAR, PR China; Universidade Federal Fluminense, BRAZIL

## Abstract

**Background:**

Mandibular setback osteotomies potentially lead to narrowing of the pharyngeal airways, subsequently resulting in post-surgical obstructive sleep apnea (OSA).

**Objective:**

To summarize current evidence from systematic reviews that has evaluated pharyngeal airway changes after mandibular setback with or without concomitant upper jaw osteotomies.

**Methodology:**

PubMed, EMBASE, Web of Science, and Cochrane Library databases were searched with no restriction of language or date. Systematic reviews studying changes in pharyngeal airway dimensions and respiratory parameters after mandibular setback with or without concomitant upper jaw osteotomies have been identified, screened for eligibility, included and analyzed in this study.

**Results:**

Six systematic reviews have been included. While isolated mandibular setback osteotomies result in reduced oropharyngeal airway dimensions, the reduction is lesser in cases with concomitant upper jaw osteotomies. Only scarce evidence exists currently to what happens to naso- and hypo-pharyngeal airways. There is no evidence for post-surgical OSA, even though some studies reported reduced respiratory parameters after single-jaw mandibular setback with or without concomitant upper jaw osteotomies.

**Conclusion:**

Although mandibular setback osteotomies reduce pharyngeal airway dimensions, evidence confirming post-surgical OSA was not found. Nevertheless, potential post-surgical OSA should be taken into serious consideration during the treatment planning of particular orthognathic cases. As moderate evidence exists that double-jaw surgeries lead to less compromised post-surgical pharyngeal airways, they should be considered as the method of choice especially in cases with severe dentoskeletal Class III deformity.

**Study registration:**

PROSPERO (registration number: CRD42016046484).

## Introduction

Little attention has been paid to mandibular setback osteotomies and potentially compromised concomitant pharyngeal airways, though sporadic cases of post-surgical obstructive sleep apnea (OSA) have been reported since the 1980s[1, 2]. This potential post-surgical complication has been investigated more vigorously only since the last two decades. Numerous researchers[3–17] have investigated and evaluated the relationship between various orthognathic procedures, concomitant changes of pharyngeal airway dimensions and OSA. Movements of mandibular jaw segments during orthognathic surgery will affect the hyoid bone and tongue positions, which in turn might influence pharyngeal airway dimensions[18]. A narrow pharyngeal airway has always been considered as a predisposing factor for OSA, a disease that might affects both patient’s quality of life and physical health[19].

In principle, mandibular prognathism can be corrected by single-jaw mandibular setback osteotomies. However, a severe sagittal antero-posterior (AP) discrepancy of the jaws is usually tackled by a concomitant upper jaw osteotomy to reduce the magnitude of the mandibular setback. To date, some authors[10, 20] have claimed that single-jaw mandibular setback osteotomies will reduce pharyngeal airway dimensions significantly, while others[6, 7] suggested that two-jaw orthognathic surgeries might produce a less compromised post-surgical pharyngeal airways.

The actual anatomical and physiological changes in post-surgical pharyngeal airways, especially in correlations with different jaw movements are yet to be established. Therefore, an overview of systematic reviews is valuable to analyze and summarize the available data, and to identify any weaknesses, inconsistencies or unanswered questions in this research field. Hence, this article aimed to summarize and analyzed critically to date’s evidence from systematic reviews regarding to the question of how mandibular setback with or without concomitant maxillary osteotomies affects the post-surgical pharyngeal airway’s dimensions and respiratory outcomes in relation to iatrogenic post-surgical OSA.

## Methodology

The reporting of these systematic reviews adheres to the Cochrane’s recommendation on overview of systematic reviews[21], and the Preferred Reporting Items for Systematic Reviews and Meta-analyses (PRISMA) statement[22, 23] where relevant. A review protocol was developed and registered with PROSPERO; registration number CRD42016046484 (http://www.crd.york.ac.uk/prospero/display_record.asp?ID=CRD42016046484).

### Search method

The electronic databases PubMed, EMBASE, Web of Science, Scopus and Cochrane Library were searched using the search strategy outlined in Table 1. The Web of Science database search has included the search of both journals and proceedings. The last search was performed on 22^nd^ April 2017 with no limitation on publication language or timeline. Subsequently, the search results were exported into Endnote X7 (Thomson Reuters, CA, USA) and duplicates were removed. The title and abstract of all articles were then screened for eligibility according to the pre-determined inclusion and exclusion criteria. The full texts of relevant articles were retrieved. Lastly, the reference lists of those articles were screen manually for further relevant articles. Two authors (TSK and RAZ) have performed both electronic and manual searches independently. Disagreement was resolved by discussion with the other two authors.

**Table 1:** Electronic databases search strategy (refer to S1 Text for the detailed search strategy).

**ELECTRONIC DATABASES**	**SEARCH STRATEGY**
**PubMed**	(Systematic review OR review OR overview OR meta-analysis OR evidence based medicine OR evidence based dentistry OR review literature OR literature review)
**EMBASE**	AND
**Web of Science**	(orthognathic surgery OR orthognathic surgical procedure OR orthodontics surgery OR mandibular surgery OR maxillary surgery OR bimaxillary surgery OR jaw surgery OR surgical orthodontic treatment OR jaw setback OR jaw movement OR mandibular setback OR maxillary advancement)
**Cochrane library**	AND
**Scopus**	(upper airway OR pharynx OR pharyngeal OR oropharynx OR oropharyngeal OR nasopharynx OR nasopharyngeal OR hypopharynx OR hypopharyngeal)

### Selection of reviews

This overview has included systematic reviews that have assessed linear, cross sectional plane, or volumetric pharyngeal airway changes related to mandibular setback with or without concomitant maxillary osteotomies. Additionally, data on respiratory parameter changes in those reviews have also been assessed.

However, systematic reviews including cleft lip and palate and/or syndromic patients as well as reviews comprising cases of distraction osteogenesis were excluded from this overview.

### Data extraction and management

Two authors (TSK, RAZ) extracted the following data from eligible systematic reviews independently: authors, publication year and title, method of analysis, number and study design of included studies, sample population (number, age and gender of patients), type of interventions, outcome measures and main findings, and follow up period.

Subsequently, all extracted data were inserted in pre-tabulated data sheets (Excel, Microsoft, New Mexico). Any disagreement was resolved by consensus of all authors to ensure consistency and reliability of extracted data.

### Assessment of methodological quality of included reviews

The methodological quality of all included reviews was assessed independently by TSK and RAZ, using the Assessment of Multiple Systematic Reviews (AMSTAR) tool[24]. Furthermore, the quality of evidence of the primary studies included in this overview was evaluated based on assessments reported by each systematic review. Disagreements were resolved by in-depth discussion among all authors.

### Data synthesis

Generally, the overview of the included systematic reviews was narrated. Additionally, meta-analysis was also performed when possible by pooling the data from primary studies across different included reviews using the “Review Manager” software (RevMan version 5.3; Copenhagen: Nordic Cochrane Center, Cochrane Collaboration; 2014). Only one primary study was included in the meta-analysis in case of an overlapping. Treatment effects across the studies were combined using the fixed effect model. The heterogeneity of trial results was assessed with the χ^2^ test for heterogeneity (p = 0.1) and the *Ι*^*2*^ measure for inconsistency. A significant heterogeneity was considered when p < 0.1 for χ^2^ test or *Ι*^*2*^ > 50%. Funnel plot was used to assess publication bias and Egger regression test was used to assess asymmetric funnel plot when more than ten primary studies were included in an analysis[25, 26].

## Results

### Quantity of current evidence

**Fig 1:**
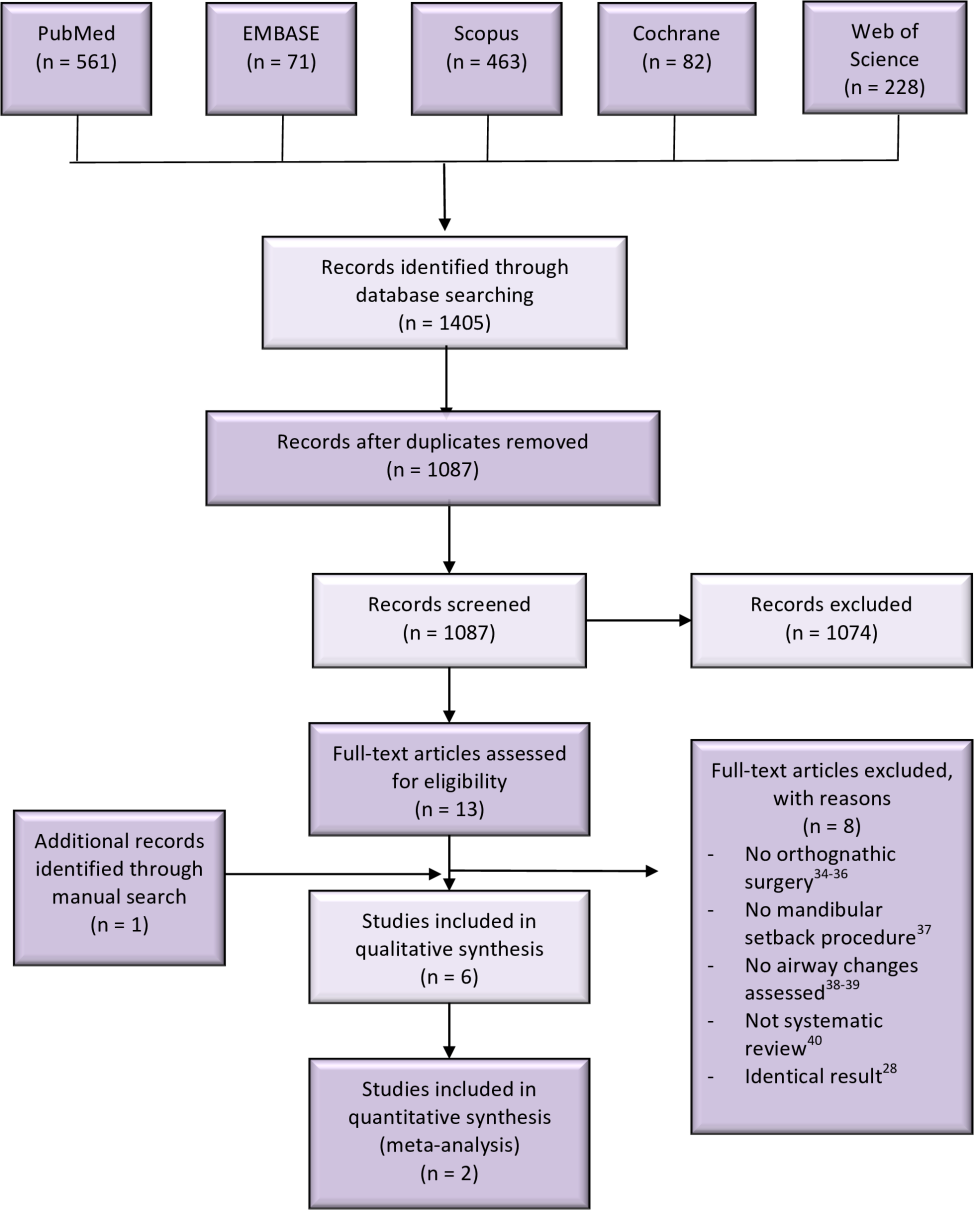
Study selection process.

The search of electronic databases has generated an overall of 1405 articles. Titles and abstracts of 1087 articles were screened after removing duplicates. Full texts of 13 relevant articles were retrieved and assessed for their inclusion eligibility. The manual search of the reference lists of those 13 articles revealed one more relevant article. Seven articles[18, 27–32] have fulfilled both inclusion and exclusion criteria. A group of authors, with identical meta-analyses and results have published two systematic reviews[27, 28] in two different languages[33]. Although there was no language limitation on article selection, only one[27] of these studies has been included in this overview due to the reason stated above. Finally, eight articles were excluded[28, 34–40], while only six articles[18, 27, 29–32] were included for further analyses. The study selection process is summarized in Fig 1.

Four[27, 29, 30, 32] of these systematic reviews have focused on mandibular setback with or without concomitant maxillary osteotomies, while two others[18, 31] investigated multiple orthognathic procedures. The characteristics of the included articles are shown in Table 2. Although five reviews[18, 27, 29–31] declared no conflict of interest, there was one review[32] did not mention about it. Besides, four included systematic reviews[18, 29, 31, 32] reported that they were not funded, one[30] did not declare on funding status, and only one review[27] was funded.

**Table 2:** Characteristics of included systematic reviews.

Authors, year	Type of review	Database searched	Primary studies that have assessed dimensional changes of upper airway									
			Airway assessed	Included studies		Participants			Interventions	Outcome	Measurement	Maximum follow-up period *(Range)*
				With mandibular setback *(Total)*	Type	Total number *(Range)*	M/F	Age *Range of mean (years)*				
Al-Moraissi *et al*[29], 2015	MA	Pubmed, Ovid, MEDLINE, Cochrane Central.	NP	8 (8)	1 RCT	380	114/266	23.3-	198 BM	Linear and cross-sectional area	6 ceph	PO 6 months-
			OP		5 CCT	(21–78)		28.2	183 MS		1 CT	2 years
			HP		2 R			*(NR*: *2 studies)*			1 CBCT	*(NR*: *3 studies)*
Canellas *et al*[30], 2015	SR	PubMed interface of Medline, Science Direct platform, Cochrane library.	PAS	9^#^ (9)	2 CCT	232	84/148	20–25	134 BM	Cross-sectional area, volume and breathing parameters	1 CBCT	PO 6–17 months
					6 CS	(6–78)		*(NR*: *1 study)*	98 MS		7 Ceph	
											1 NR	
Christovam *et al*[31], 2016	MA	Cochrane library, Medline (via PubMed), Scopus, VHL, Web of Science, Open-Grey.	UA	15 (27)	13 R	391	185/181	20.3–30.04	106 MS	Cross-sectional area and volume	CT	PO 2 months-
									781 MxA+MS			
					2 P	(12–60)	*(NR*: *1 study)*		30 MxI+MS			>1 year
									30 MxS+MxI+MS			
									20MxS+Ms			
Fernandez-Ferrer *et al*[32], 2015	SR	Scopus, Pubmed, Cochrane, EMBASE.	NP	14 (14)		369	160/197	20.3–25.5	MS	Linear, cross-sectional area, volume and respiratory parameters	8 3-D imaging	28 days-1.4 years
			OP		12 R							
					2 P	(9–78)	*(NR*: *2 studies)*	*(NR*: *3 studies)*	BM		6 ventilation	*(NR*: *2 studies)*
			HP									
He *et al*[27], 2017	MA	Scopus, Pubmed, Cochrane, EMBASE, Web of Science	NP	18 (18)		564	253/311	18.8–26.6	299 MxA+MdS	Cross-sectional area and volume	CT	NR
			OP		12R							
					6P	(11–72)			264 Mds			
			HP									
Mattos *et al*[18], 2011	MA	VHL, Scirus, Ovid, SIGLE.	OP	19(22)	12 R	665	107/426	17.9–30	428 MS	Linear and cross-sectional area	15: Ceph	PO 1
					7 P	(10–66)	*(NR*: *3 studies)*	*(NR*: *1 study)*	205 MxA+MS		2: CT	month-
											2: Ceph + CT	12 years

^#^ One of the included study did not assess the upper airway changes, but post-operative OSA was evaluated

Abbreviations:

SR = systematic review; MA = meta-analysis; NP = nasopharyngeal, OP = oropharyngeal, HP = hypopharyngeal, UA = upper airways, PAS = pharyngeal airway space; R = Retrospective study; P = Prospective study; RCT = randomized controlled trial; CCT = Case controlled trials; CS = Case series; MS = Mandibular setback, BM = bimaxillary surgery; MxA = Maxillary advancement, MMA = Maxillomandibular advancement; MxI = Maxillary impaction; MxS = maxillary; setback; Ceph = Cephalometric; CT = Computed tomography; CBCT = Cone-beam computed tomography; NR = not reported; PO = post-operative

### Quality of systematic reviews (AMSTAR)

Analysis with the AMSTAR tool revealed that only three systematic reviews with high scores of 8[27, 31] and 10[18] “yes”, whereas the others[29, 30, 32] have scored 5 or less (Table 3). Although only three systematic reviews[18, 27, 31] reported on an ‘a priori’ design, all six articles have performed a comprehensive literature search with three or more electronic databases. The review of Mattos *et al*[18] accomplished the highest score of “yes”, and was the only review that has listed and referenced both included as well as excluded studies.

**Table 3:** Quality assessment of included systematic reviews with AMSTAR tool.

AMSTAR criteria	*Al-Moraissi *et al*[29]	Canellas *et al*[30]	*Christovam *et al*[31]	Fernandez-Ferrer *et al*[32]	He *et al*[27]	*Mattos *et al*[18]
1. Was an ‘a priori’ design provided?	CA	CA	Y	CA	Y	Y
2. Was there duplicate study selection and data extraction?	CA	Y	Y	Y	Y	Y
3. Was a comprehensive literature search performed?	Y	Y	Y	Y	Y	Y
4. Was the status of publication (i.e. grey literature) used as an inclusion criterion?	Y	N	Y	N	N	Y
5. Was a list of studies (included and excluded) provided?	N	N	N	N	N	Y
6. Were the characteristics of the included studies provided?	Y	Y	Y	Y	Y	Y
7. Was the scientific quality of the included studies assessed and documented?	Y	N	Y	Y	Y	Y
8. Was the scientific quality of the included studies used appropriately in formulating conclusions?	N	NA	Y	N	Y	Y
9. Were the methods used to combine the findings of studies appropriate?	Y	N	Y	Y	Y	Y
10. Was the likelihood of publication bias assessed?	N	N	N	N	Y	Y
11. Was the conflict of interest stated?	CA	CA	CA	CA	CA	CA
TOTAL “YES”	5	3	8	5	8	10

Y = yes; N = no; CA = can’t answer; NA = not applicable

* Systematic reviews with meta-analysis

### Quality of evidence from primary studies in included reviews

The majority of primary articles were retrospective studies with only one randomized controlled trial (Table 2). While Canellas *et al*[30] did not report on quality assessment of their included primary studies, no uniform quality assessment tool was used to assess the quality of primary studies across the other five systematic reviews. Christovam *et al*[31] have applied the assessment criteria compiled by Mattos *et al*[18] with a different scoring system that they have developed on their own (Table 4).

**Table 4:** Quality assessment for primary studies of included systematic reviews.

QUALITY ASSESSMENT	SYSTEMATIC REVIEWS					
	Al-Moraissi *et al*[29]	Canellas *et al*[30]	Fernandez-Ferrer *et al*[32]	He *et al*[27]	Christovam *et al*[31]	Mattos *et al*[18]
Assessment method*	Self-developed criteria to assess risk of bias (based on MOOSE, STROBE and PRISMA)	Not reported	CONSORT criteria	MINORS criteria	Risk of bias based on quality assessment method reported by Mattos *et al*[18]	Self-compiled criteria for quality of methodological soundness (mostly based on CONSORT statement)
Assessment criteria	1. Random selection in population 2. Definition of inclusion and exclusion criteria 3. Report of losses to follow-up 4. Validated measurements 5. Statistical analysis	-	Not reported	12 items (details not reported)	1. Eligible criteria for participants described 2. Presence of control group 3. Blinding assessment stated 4. Statistical treatment performed 5. Reliability of measures tested 6. Reporting drop-outs 7. Follow-up period reported 8. Potential bias and trial limitations addressed	
Scoring method	Low risk (included all criteria), moderate risk (did not include one of the criteria), high risk (two /> criteria were missing)	-	Not reported	Low risk of bias (19–24); Moderate risk (13–18); High risk (0–12)	Low risk of bias (≥4.5); Moderate risk (>2 and <4.5); High risk (≤2)	High quality (>6 points); Moderate quality (4–6 points); Low quality (<4 points)
Results	1 low risk; 7 moderate risk	-	11 moderate quality; 3 high quality	8 low risk; 10 moderate risk	6 low risk; 7 moderate risk;	11 moderate quality
Remark	-	-	Refer to text in discussion	-	High risk paper was excluded from the review	Low quality studies were excluded from the review.

* MOOSE: Meta-Analysis of Observational Studies in Epidemiology Statement; STROBE: Strengthening the Reporting of Observational Studies in Epidemiology statement; PRISMA: Preferred Reporting items for Systematic Reviews and Meta-Analyses; CONSORT: Consolidated Standards of Reporting Trials; MINORS: Methodological Index for Non-Randomized Studies

Not all primary studies have been analyzed quantitatively in the four included meta-analyses[18, 27, 29, 31]. Hence, this section only evaluated primary studies that have been included and analyzed in those systematic reviews. Out of the 64 included primary studies being assessed, 18 were rated with a high quality or low risk of bias, while others were rated with moderate quality or risk of bias.

### Airway changes in linear measurements

#### 1. Nasopharyngeal (NP) airway

Al-Moraissi *et al*[29] did not find any significant differences (*p* = 0.72) in the antero-posterior (AP) dimension of post-surgical NP airways when comparing two-jaw versus single-jaw mandibular setback surgeries, using random-effects modeling in their meta-analysis (I^2^ = 78%); MD = 0.11mm [95% CI -0.49, 0.71]; 264 patients in five studies (172 double-jaw; 90 single jaw).

#### 2. Oropharyngeal (OP) airway

Mattos *et al*[18] performed multiple meta-analyses to evaluate post-surgical OP airway changes based on different measurement locations after mandibular setback with or without concomitant maxillary osteotomies (Table 5). All results showed significantly reduced post-surgical AP dimension, except for the measurement from the posterior nasal spine to the pharyngeal wall that increased significantly (*p*<0.00001).

**Table 5:** Anteroposterior (AP) changes of OP airway at multiple measurement locations (based on meta-analyses results reported by Mattos *et al*[18]).

**Meta-analyses**	**Measurement location**	**Type of surgery**	**Number of primary studies**	**Number of patients**	**Result**
					*(AP dimension of OP airway)*
**1**	PNS-pharyngeal wall	Maxillary advancement + mandibular setback	3	62	Significant increase (*p*<0.00001). MD = 3.81mm [95% CI 2.46, 5.16], I^2^: 0%,
**2**	Soft palate-pharyngeal wall	Mandibular setback	5	142	Significant decrease (*p*<0.00001). MD = -2.57mm [95% CI -3.3, -1.85], I^2^ = 50%
		Maxillary advancement + mandibular setback	6	159	Significant decrease (*p* = 0.01). MD = -0.91mm [95%CI -1.62, -0.20], I^2^ = 69%
**3**	Base of tongue-pharyngeal wall	Mandibular setback	7	190	Significant decrease (*p*<0.00001). MD = -2.99mm [95% CI -3.67, -2.31], I^2^ = 35%
		Maxillary advancement + mandibular setback	2	43	Significant decrease (*p*<0.00001). MD = -2.83mm [95%CI -3.98, -1.68], I^2^ = 0%
**4**	Vellacula-pharyngeal wall	Maxillary advancement + mandibular setback	3	63	Significant decrease (*p*<0.0001). MD = -2.20mm [95% CI -3.23, -1.18], I^2^ = 0%

PNS = posterior nasal spine

Another meta-analysis[29] of five studies compared two-jaw (maxillary advancement and mandibular setback osteotomies) versus mandibular setback surgeries in 253 patients (152 two-jaw; 101 one-jaw). A highly significant difference in the post-surgical AP dimension was found favoring two-jaw over one-jaw surgeries (*p*<0.00001) in OP airways; MD = 3.04mm [95%CI2.76, 3.32], I^2^ = 48%.

#### 3. AP measurement at minimal pharyngeal airway space

One meta-analysis[18] analyzing mandibular setback combined with maxillary advancement osteotomies discovered no significant changes related to the post-surgical minimal pharyngeal airway space (*p* = 0.72); MD = 0.27mm [96% CI -1.19, 1.72], I^2^ = 0%, 2 studies, 63 patients.

#### 4. Lateral width of the oropharyngeal (OP) airways

A significantly decreased (*p* = 0.01) lateral width of OP airways at the level of the tongue base was detected after mandibular setback osteotomies; MD = -2.37mm [95% CI -4.23, -0.51], I^2^ = 0%, 2 studies, 54 patients.[18]

#### 5. Bilateral sagittal split osteotomy (BSSO) versus vertical subsigmoid osteotomies (VSSO)

In a total of 69 patients (42 BSSO, 27 VSSO), two studies (1 randomized controlled trial, 1 retrospective study) investigated the effects of two different setback procedures on anteroposterior OP airway dimension by means of cephalometric analysis. The meta-analysis disclosed a highly significant (*p* = 0.006) narrower post-surgical OP airways after VSSO compared to BSSO; MD = 1.29mm [95% CI 0.37, 2.22], I^2^ = 0%.[29]

### Cross-sectional airway changes

#### 1. Nasopharyngeal (NP) airway

Al-Moraissi *et al*[29] pooled the results from three studies with an overall of 109 dentoskeletal class III patients (64 two-jaw, 45 single-jaw osteotomies) and compared the cross-sectional plane changes associated with each procedure. They concluded that maxillary advancement combined with mandibular setback osteotomies provide more favorable results than mandibular setback only (*p* = 0.0004); MD = 0.76mm^2^ [95% CI 0.34, 1.18].

This result was supported by another meta-analysis[27] of four studies (54 two-jaw, 63 single-jaw osteotomies) that also favored two-jaw over one-jaw surgeries (*p =* 0.002); MD = -0.59mm^2^ [95% CI -0.97, -0.22]; I^2^ = 0%.

#### 2. Oropharyngeal (OP) airway

Al-Moraissi *et al*[29] further analyzed quantitatively cross-sectional plane changes of OP airways comparing two-jaw versus mandibular setback surgeries. Data evaluation from three studies comprising 109 patients (64 two-jaw, 45 single-jaw) revealed two-jaw surgeries result in more favorable post-surgical cross-sectional dimension (*p* = 0.01), MD = 1.37mm^2^ [95% CI 0.27, 2.46]; I^2^ = 82%. However, regional analysis at the level of soft palate have shown no significant difference between one- versus two-jaw surgery (*p =* 0.05) in six studies (107 two-jaw; 98 one-jaw); MD = -0.28mm^2^ [95% CI -0.57, 0.00]; I^2^ = 0%.[27].

Other regional meta-analyses of two-jaw surgeries have discovered no significant difference upon comparison of pre- and post-surgical OP airways at the level of soft palate (*p* = 0.59), MD = -10.73mm^2^ [95% CI -49.53, 28.07]; I^2^ = 0%, and tongue base (*p* = 0.36), MD = -16.88mm2 [95% CI -53.21, 19.44]; I^2^ = 0%.[18] The same authors[18], however, disclosed a highly significant (*p* = 0.004) reduction of post-surgical cross-sectional plane at the level of the tongue base after isolated mandibular setback osteotomies, MD = -46.23mm2 [95%CI -77.51, -14.96]; I^2^ = 0%.

#### 3. Hypopharyngeal (HP) airway

One meta-analysis[29] investigated differences in HP airway changes after one- versus two-jaw surgeries in dentoskeletal class III patients. Based on one cephalometric and one CBCT studies, MD = 3.04mm^2^ [95% CI -2.15, 8.23], I^2^ = 97%, no significant difference (*p* = 0.25) between both procedures related to post-surgical cross-sectional HP airway changes was disclosed.

On the contrary, He *et al*[27] reported a highly significant result at the level of the epiglottis plane in post-surgical cross-sectional area favoring two-jaw over one-jaw surgeries (*p* = 0.002) in 6 studies (107 two-jaw, 98 one-jaw); MD = -0.46mm^2^ [95% CI -0.75, -0.17], I^2^ = 0%.

#### 4. Regional minimum cross sectional area (CSA^min^)

At the retro-palatal level, a significant increase of CSA^min^ was found after both two-jaw (118.63mm^2^) and one-jaw (23.03mm^2^) surgeries[31]. Meanwhile, two-jaw surgeries were also found to significantly increase the CSA^min^ (94.84mm^2^) at the retrolingual level.[31]

### Volumetric airway changes

Total volumetric changes have been assessed in two meta-analyses[27, 31]. Christovam *et al*[31] reported a significant decrease of the total airway volume after mandibular setback osteotomies (*p = 0.00, mean = -1894.67mm^3^, six studies, 106 patients), as well as after combined maxillary advancement and mandibular setback osteotomies (*p = 0.00, mean = -1552.91mm^3^, 11 studies, 187 patients). However, no significant difference could be found when comparing one- versus two-jaw surgeries (*p* = 0.067, 3 studies, 97 patients, 54 single-jaw surgery, 43 double-jaw surgery).[31]

In contrast, He *et al*[27] have indicated that two-jaw surgeries are more favorable than one-jaw surgeries (*p =* 0.002) after assessing the post-surgical changes of total pharyngeal airway volume in four studies (75 two-jaw, 62 one-jaw); MD = -3.41ml, 95% CI -5.59, -1.29; I^2^ = 0%. However, their detailed analyses of regional volumetric changes indicated that the significant result favoring two-jaw surgeries only occurred at the level of NP (*p<*0.0001), but not at the level of OP (*p =* 0.08) or HP (*p =* 0.64)[27].

* An exact p-value was not revealed in the article.

**Fig 2:**
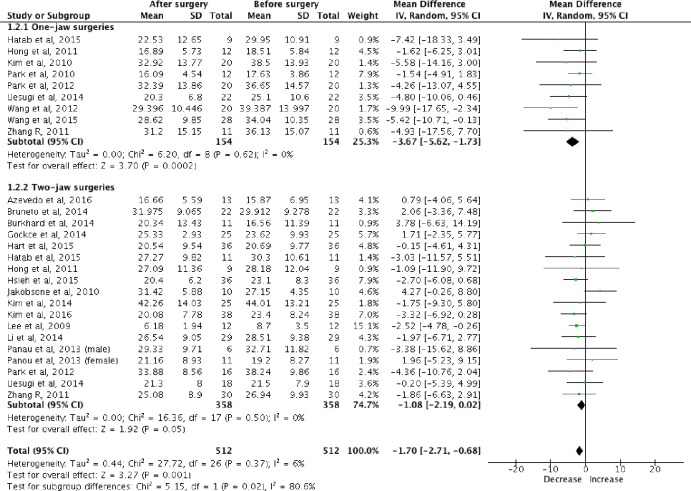
Total volumetric changes of pharyngeal airway after mandibular setback (one-jaw) and mandibular setback with maxillary advancement (two-jaw) surgeries.

A meta-analysis was performed combining the primary studies of these two systematic reviews[27, 31] assessing the post-surgical total volumetric changes for pharyngeal airways, in one-jaw and two-jaw surgeries (Fig 2). Mandibular setback surgeries were found to significantly reduce (*p* = 0.0002) the post-surgical total pharyngeal airway volume (mean = -3.67ml, nine studies, 154 patients). On the contrary, no significant difference (p = 0.05) was detected in total pharyngeal airway volume after mandibular setback with maxillary advancement surgeries.

**Fig 3:**
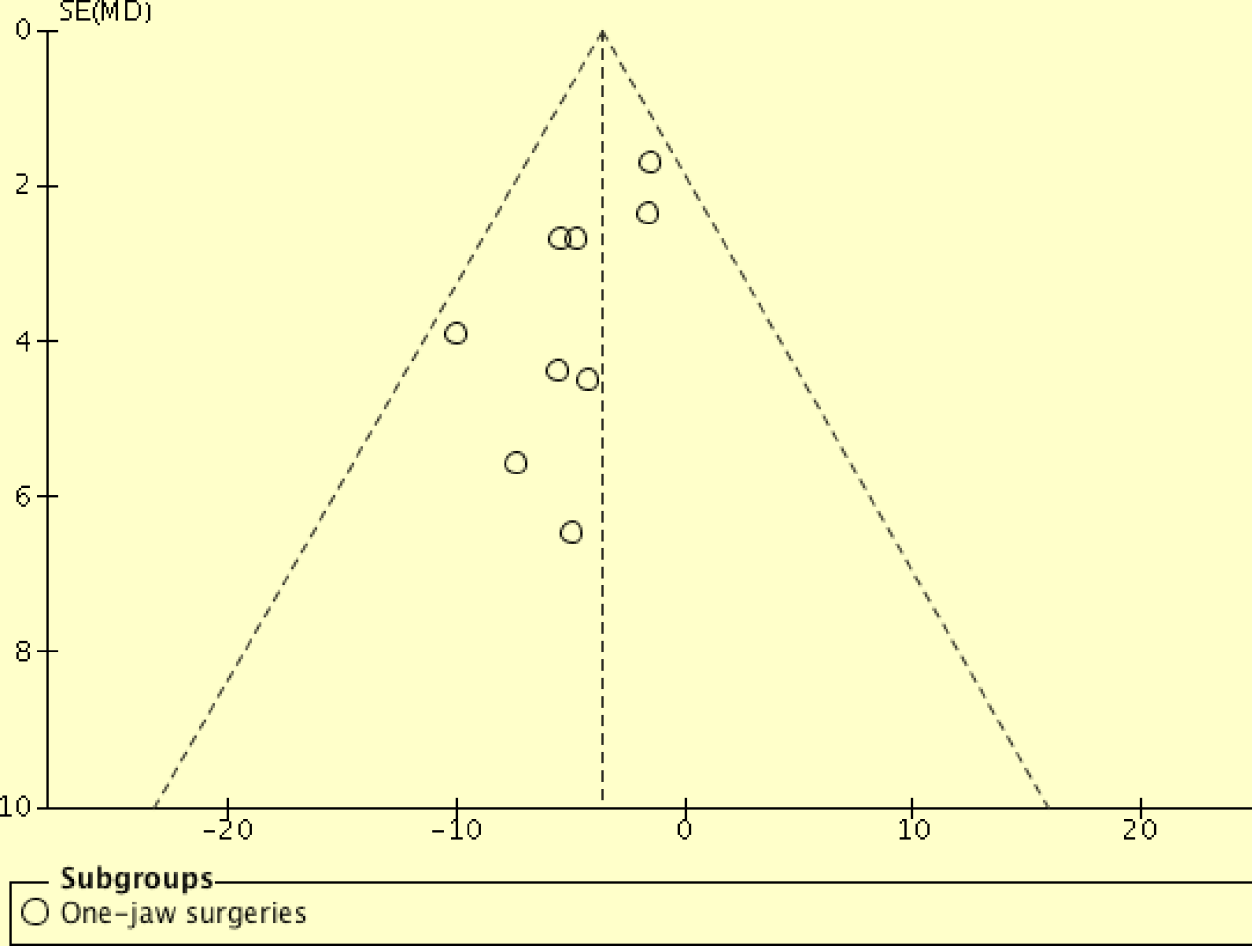
Funnel plot for primary studies of mandibular setback surgeries.

**Fig 4:**
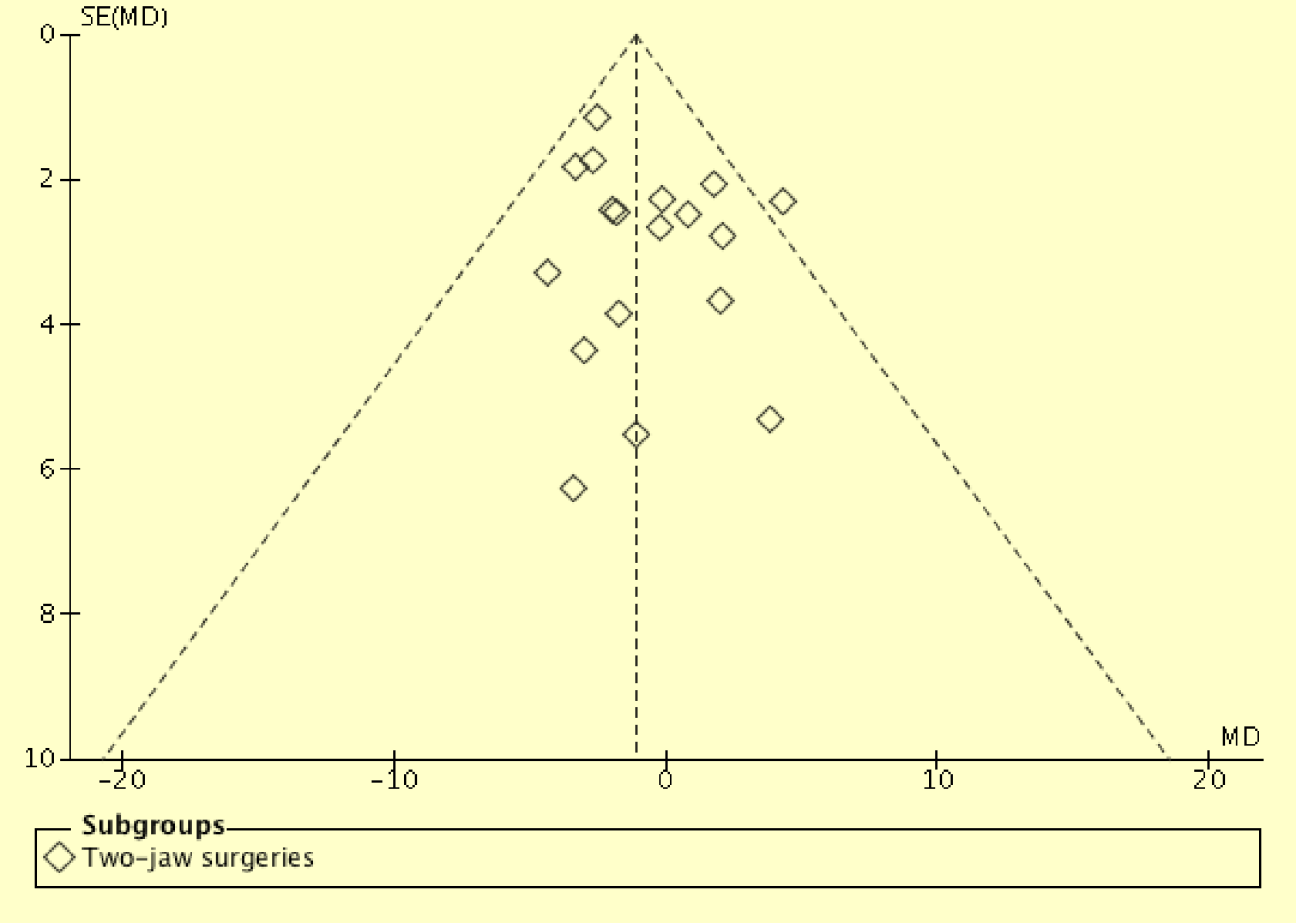
Funnel plot for primary studies of mandibular setback with maxillary advancement surgeries.

Additionally, one-jaw surgeries were found to reduce post-surgical total pharyngeal airway volume significantly (p = 0.02) compared to two-jaw surgeries (154 one-jaw; 358 two-jaw). This one-jaw versus two-jaw comparison has involved a much larger sample size (512 patients) compared with Christovam *et al*[31] (97 patients) and He *et al*[27] (137 patients). Assessments with funnel plots were performed (Figs 3 and 4). Asymmetric funnel plot was found for mandibular setback surgeries (Fig 3). However, Egger regression test was not performed, as tests for funnel plot asymmetry were not recommended when there are fewer than 10 studies in the analysis[26]. This asymmetry might be resulted from reporting bias, poor methodology quality in smaller studies, true heterogeneity, artifactual or by chance[26]. Future meta-analyses should investigate the cause for the funnel plot asymmetry when more primary studies are available.

### Respiratory outcome measures

Both Canellas *et al*[30] and Fernandez-Ferrer *et al*[32] did not perform statistical meta-analyses in their systematic reviews. The former[30] included nine studies (232 patients) investigating the respiratory parameters in relation to one- or two-jaw surgeries.[30] Only seven patients showed an increased Apnea-Hypopnea Index (AHI) and Oxygen Desaturation Index (ODI) post-surgically, while no respiratory parameter deterioration was found among the others.[30] Both significant AHI and ODI findings after two-jaw surgeries were reported in the same primary study[41].

Canellas *et al*[30] have also detected significant but contradicting results of arterial oxygen saturation (SpO_2_) in two single-jaw surgery studies: one[8] with significantly reduced, the other[17] with significantly increased values. Meanwhile, Fernandez-Ferrer *et al*[32] detected that no post-surgical SpO_2_ reduction or AHI changes persisted in the long term.

### Obstructive sleep apnea (OSA)

Canellas *et al*[30] reported that two out of 232 patients have developed post-surgical mild OSA. Both cases occurred after large mandibular setback movements of 13.7mm (single-jaw surgery) and 12.6mm (double-jaw surgery), respectively measured from the pogonion[30]. There were two systematic reviews[30, 32] screened the literature for respiratory parameters. Both concluded that 1) no evidence to confirm OSA development after mandibular setback[30, 32] or two-jaw[32] osteotomies within the first six months after surgery[30] and 2) respiratory changes do not persist in the long term[32].

## Discussion

### Impact of mandibular setback osteotomies on pharyngeal airways

Most primary studies of the included systematic reviews assessed pharyngeal airway changes by means of 2-D cephalometric analyses. As this technique allows only linear measurements, it cannot accurately assess 3-dimensional pharyngeal airway changes. Another significant limitation inherent to most studies was a lack of information about the head and neck posture and the tongue position during imaging[18]. Though RCTs stand for the highest level of evidence among clinical studies, under many circumstances it is neither ethical nor practical to perform RCTs to study various elective orthognathic techniques and their effect on airway changes. Therefore, prospective clinical studies with 3-D imaging and a defined long-term follow-up might probably represent the most appropriate future study design for this research topic.

Reports about nasopharyngeal (NP) airway changes after mandibular setback osteotomies are rare. While no significant difference was noted upon comparison of 1-jaw versus 2-jaw surgeries in AP dimension[29], interestingly 2-jaw surgery was found to generate more favorable outcomes in both cross-sectional[27, 29] and volumetric[27] analyses. It might be hypothesized that the maxillary advancement in 2-jaw surgeries possibly compensates the effect of the mandibular setback at nasopharynx.

More reports were found related to oropharyngeal (OP) airway changes associated with mandibular setback osteotomies. Generally, it was accepted that mandibular setback with or without concomitant maxillary osteotomies can lead to significantly reduced OP airway in AP dimension. An unusual AP increase of the OP airway at the level between posterior nasal spine and pharyngeal wall after two-jaw surgeries[18] probably represents the effect of concomitant maxillary advancement movement. Regional analyses[18, 27, 29] have shown more complex results indicating that post-surgical OP changes were not uniform, but differed depending on the level of measurements. Taking into account the various anatomical measurement points and methods of different authors, current results are subject to critical review.

Results of 3-D volumetric pharyngeal airway analyses seem to underlie interpretations that are more complicated. The overall pharyngeal airway volume was decreased both after 1- and 2-jaw surgeries.[31] While 1-D and 2-D analyses[29] have shown that two-jaw surgeries produced more favorable post-surgical effects on OP airways than 1-jaw surgeries, 3-D analyses of previous meta-analyses[27, 31] have reported contradictory results. However, the meta-analysis of this overview with larger sample size has supported the result of He *et al*[27] that 1-jaw surgeries resulted in a significantly reduced total pharyngeal volume in compared to 2-jaw surgeries. Again, regional analyses[27] have shown that both surgeries have different effects on the pharyngeal airways depending on the particular measurement location. Surprisingly one systematic review[31] has reported a reduction of the retro-palatal volume with an increased retro-lingual volume after 2-jaw surgeries. Even though those findings might be explained due to the novel anatomical positioning of hard and soft tissues, they are somewhat unexpected warranting further investigations, probably best in combination with dynamic airway flow measurements. 2-D and/or 3-D imaging techniques only provide static analyses of post-surgical hard and soft tissue positions and relations. The true physiological impact of new anatomical hard and soft tissue positions and their impact on dynamic airflow aspects still require in depth investigations and evaluations.

VSSO setback osteotomies resulted in significantly reduced OP airways in AP dimension compared to BSSO[29]. Posterior rotation of the mandible after releasing the mandibulo-maxillary fixation (MMF) after VSSO and post-surgical relapse in BSSO might explain this finding.[29]

Although mandibular setback osteotomies reduce OP airways to certain degree, the included reviews[30, 32] did not provide evidence that OSA develops post-surgically. Although reports of mild OSA after mandibular setback osteotomies are rare[30], nevertheless, these reports suggested that it might occur. Therefore it must not be disregarded completely as a potential adverse event after orthognathic setback osteotomies. Two-jaw surgeries should be taken into serious consideration during the treatment planning, especially in cases with severe mandibular prognathism or patients with predisposing factors for OSA[6, 7, 29, 30].

Although various articles have studied the effect of mandibular setback osteotomies on pharyngeal airways, it is difficult to find a common denominator to compare those results across the studies due to different measurement locations, methods and definitions[18, 31, 32]. For example, Al-Moraissi *et al*[29] have categorized the cross-sectional measurement of a primary study [7] in their meta-analysis at the level of soft palate (level at the most superior anterior point of the second cervical spine parallel to the Sella-Nasion line) under nasopaharyngeal (NP) group. Besides, this meta-analysis[29] has also categorized measurement at the level of posterior nasal spine (PNS) of another primary study[9] under NP. The result of this analysis should be interpreted with caution, as PNS is commonly used as the inferior border of NP and superior border of OP and therefore difficult to justify the usage of PNS to represent NP airways. Obtaining a generally accepted consensus about the most accurate and suitable pharyngeal airway measurement locations might lead to more consistent and comparable results across the studies, and ultimately to more valid evidence in the future. Christovam *et al*[31] have suggested that future studies should focus on a minimum CSP as it is not inferred by regional mean values.

None of the included reviews[18, 29–32] studied any gender related post-surgical pharyngeal airway changes, even though a few of their included primary studies displayed statements about gender related differences. While some[42, 43] could not find any gender related differences at all, others did[15, 44]. A recommendation to perform gender related subgroup analyses in the future might be taken into consideration. The maximum follow-up periods of primary studies vary but many were too short to demonstrate the eventual pharyngeal airway changes after mandibular setback surgery. This could have provided a false negative result on the incidence of post-surgical OSA. Long-term follow-up of at least 2 years post-surgically might be suitable to take into account relapse tendencies after orthognathic surgery.

It would be interesting to study effects of various simultaneous orthognathic procedures on pharyngeal airways. Concomitant orthognathic procedures such as genioplasty and maxillary impaction and their post-surgical impact on pharyngeal airways have not yet been reported adequately in primary studies. Furthermore, mandibular setback techniques (e.g. VSSO or BSSO), the extent of jaw setback movements, the patients’ BMI and the pre-existing history of snoring or other OSAS features are often neglected in patient assessments. The surgeons could then apply such additional clinical information to develop a holistic patient management. Moreover, unreported pre-surgical information of these potential clinical confounders might lead to errors in the interpretation of final treatment outcomes.

Christovan *et al*[31] have reported that two groups of authors that each has potentially duplicated their results in two different papers[45–48]. This finding was not able to be confirmed as the authors were not accessible[31]. It is very important to identify possible duplicate results during systematic reviews or meta-analyses, as otherwise the false negative or positive results might be reported. On the other hand, the reporting bias is equally important and can yield the same effect to the result of a review too. Although an asymmetry funnel plot was detected in this overview, no asymmetry test for publication bias was performed to prevent misleading the readers about false positive or negative publication bias[25].

### Quality assurance in systematic reviews

The AMSTAR[24] assessment revealed a high methodology quality in only half of the here included systematic reviews. In addition to self-declaration, systematic reviews also need to indicate funding or supportive sources for each of their primary papers; this item of the checklist was not fulfilled in any of the included systematic reviews. Besides, only He *et al*[27] and Mattos *et al*[18] have discussed the publication bias of their included primary studies, albeit the latter have failed to present a funnel plot in their article.

Language bias might represent another potential study design flaw. Any language restriction might lead to overlook of well suitable studies written in other languages, resulting in a restricted number of articles and analyzed data. Three of the here included systematic reviews[18, 29, 30] limited their search to English literature only, while others[27, 31, 32] did not mention anything about it. Besides, only one review[18] presented a reference list for both included and excluded articles. Others[27, 29–32] referenced only their included articles, another common study flaw of systematic reviews that prevents their reproducibility.

The quality of included primary studies affects directly the quality of each systematic review. Therefore, it is mandatory to assess the quality and/or risk of bias of all included primary studies. One systematic review[30] omitted the evaluation of both issues, most likely because of using the PRISMA Equity 2012[49] instead of the standard PRISMA[23] checklist. The former[49] should only be applied in systematic reviews focusing on health equity, which, however, is not applicable for this topic. Therefore, conclusions of that article should be considered with care. Others [32] have claimed that they have used the CONSORT 2010[50] guideline to assess their included twelve retro- and two prospective primary studies qualitatively. However, they did not provide any explanation on how the included studies were classified into low, medium and high quality. The CONSORT 2010[50] checklist was exclusively developed to evaluate the quality of clinical randomized controlled trials (RCT), hence, it has to be considered less appropriate for non-RCT primary studies. Applying inappropriate assessment tools in systematic reviews might further confuse the readers related to the quality of included primary studies. For example, one retrospective primary study[51] was classified with a moderate quality, even though 1) the number of cases for each procedure, 2) the follow-up period, and 3) the demographic details of patients, like gender and age were not reported.

Among the here presented systematic reviews, no standardized quality assessment tool was used. While the Cochrane Risk of Bias Tool is well-known for RCTs, so far, none has been established for non-RCTs. Inconsistent nomenclature for non-randomized studies, and taxonomies used for differently defined, but similar study designs[52] may further bedazzle researchers in their attempts to classify non-randomized trials. Subsequently, this confusion may complicate the selection of the most appropriate assessment tool. Some researchers[52, 53] have performed comprehensive searches and analyzed quality assessment tools for non-randomized clinical studies or tools that can be used to assess more than one study design. Their recommendations e.g. the Methodological Index for Nonrandomized Studies (MINORS) tool and Thomas tool might be considered useful for future systematic reviews.

The frequently applied GRADE guideline[54] was not used in the here presented overview, as it was developed to address questions related to alternative management strategies, interventions, or policies, but not for questions related to risk or prognosis[54].

## Conclusion

Mandibular setback osteotomies cause reduced pharyngeal airway dimensions. Although it has been reported sporadically, evidence that confirms the development of post-surgical OSA was not found. Nevertheless, this potential post-surgical hazard should be taken into serious consideration during the treatment planning of particular orthognathic cases. As moderate evidence exists that double-jaw surgeries may have lesser effect on post-surgical pharyngeal airways, they should be taken into consideration as the method of choice especially in cases with severe dentoskeletal Class III deformities.

## Supporting information

S1 Text Detailed search strategy. (PDF)

S2 Text Citation Matrix. (PDF)

S1 Table PRISMA checklist. (PDF)
