# Supplementary material for: Effects of mandibular setback with or without maxillary advancement osteotomies on pharyngeal airways: An overview of systematic reviews
Source: PLoS One. 2017 Oct 9;12(10):e0185951. doi: 10.1371/journal.pone.0185951 (PMC5633244; doi:10.1371/journal.pone.0185951)
Supplement: S2 Text — (PDF) [file pone.0185951.s002.pdf]

S2 Text. Citation matrix

The citation matrices presented below have only included the primary papers that were analysed quantitatively in the four included meta-analyses of this overview to avoid false positive results. The following primary papers have not been included here:

- primary papers of systematic reviews without meta-analysis
- primary papers not quantitatively analysed in systematic review with meta-analysis

The corrected covered area (CCA)[1] was calculated separately for different measurement analyses based on own citation matrix to represent the actual overlapping situation. However, CCA was not calculated for the measurement analyses reported by only one systematic review.

Formula of CCA[1]: CCA= (N-r)/(rc-r)

N= number of included pulications (including double counting)
r= number of rows (number of index publications)
c= number of columns (number of reviews)

Interpretation of CCA scores[1]:

0-5 : slight overlap
6-10: moderate overlap
11-15: high overlap
>15: very high overlap

Table 1. Citation matrix of primary papers related to antero-posterior dimension measurement of pharyngeal airway after mandibular setback surgery

		**Mattos *et al*, 2011[2]**	**Al-Moraissi *et al*, 2015[3]**
**Primary papers**	**Soft palate-pharyngeal wall (MdS)**		
	Chen et al, 2007[4]	/	
	Degerliyurt et al, 2008[5]	/	
	Liukkonen et al, 2002[6]	/	
	Muto et al, 2008[7]	/	
	Park et al, 2010[8]	/	
	**Soft palate-pharyngeal wall (MdS+ MxA)**		
	Cakarne et al, 2003[9]	/	
	Chen et al, 2007[4]	/	
	Degerliyurt et al, 2008[5]	/	
	Jakobson et al, 2010[10]	/	
	Marsan et al, 2009[11]	/	
	Mehra et al, 2001[12]	/	
	**Base of tongue-pharyngeal wall (MdS)**		
	Chen et al, 2005[13]	/	
	Degerliyurt et al, 2008[5]	/	
	Kawakami et al, 2005[14]	/	
	Kawamata et al, 2000[15]	/	
	Liukkonen et al, 2002[6]	/	
	Muto et al, 2008[7]	/	
	Park et al, 2010[8]	/	
	**PNS-pharyngeal wall (MdS+MxA)**		
	Cakarne et al, 2003[9]	/	

	Chen et al, 2007[4]	/	
	Jakobson et al, 2010[10]	/	
	**Base of tongue-pharyngeal wall (MdS+MxA)**		
	Degerliyurt et al, 2008[5]	/	
	Mehra et al, 2001[12]	/	
	**Vallecula-pharyngeal wall (MdS+MxA)**		
	Cakarne et al, 2003[9]	/	
	Chen et al, 2007[4]	/	
	Jakobson et al, 2010[10]	/	
	**At minimal pharyngeal space (MdS+MxA)**		
	Jakobson et al, 2010[10]	/	
	Marsan et al, 2009[11]	/	
	**Nasopharyngx (1-jaw vs 2- jaw)**		
	Chen et al, 2007[4]		/
	Hwang et al, 2010[16]		/
	Hong et al, 2011[17]		/
	Aydemir et al, 2012[18]		/
	Kobayashi et al, 2013[19]		/
	**Oropharynx (1-jaw vs 2- jaw)**		
	Chen et al, 2007[4]		/
	Degerliyurt et al, 2008[5]		/
	Hong et al, 2011[17]		/
	Aydemir et al, 2012[18]		/
	Kobayashi et al, 2013[19]		/

MdS= Mandibular setback
MxA= Maxillary advancement

Table 2. Citation matrix of primary papers related to cross-sectional area (CSA)measurement of pharyngeal airway after mandibular setback surgeries

		**He *et al*, 2017[20]**	**Mattos *et al*, 2011[2]**	**Al-Moraissi *et al*, 2015[3]**
**Primary papers**	**Min CSA (MdA + MxA)**			
	Hatab et al, 2015[21]	/		
	Azevedo et al, 2016[22]	/		
	Hart et al, 2015[23]	/		
	Kim et al, 2016[24]	/		
	Hsieh et al, 2015[25]	/		
	**Level of posterior nasal spine (1-vs 2- jaw)**			
	Hatab et al, 2015[21]	/		
	Hong et al, 2011[17]	/		
	Park et al, 2012[26]	/		
	Uesugi et al, 2014[27]	/		
	**Level of soft palate (1-vs 2- jaw)**			
	Hatab et al, 2015[21]	/		
	Hong et al, 2011[17]	/		
	Zhang R, 2011[28]	/		
	Park et al, 2012[26]	/		
	Uesugi et al, 2014[27]	/		
	Degerliyurt et al, 2008[5]	/		
	**Level of epiglottis (1-vs 2- jaw)**			
	Hatab et al, 2015[21]	/		
	Hong et al, 2011[17]	/		
	Zhang R, 2011[28]	/		
	Park et al, 2012[26]	/		
	Uesugi et al, 2014[27]	/		

	Degerliyurt et al, 2008[5]	/		
	**Level of nasopharynx (1- vs 2-jaw)**			
	Degerliyurt et al, 2008[5]			/
	Hong et al, 2011[17]			/
	Aydemir et al, 2012[18]			/
	**Level of oropharynx (1- vs 2-jaw)**			
	Degerliyurt et al, 2008[5]			/
	Hong et al, 2011[17]			/
	Aydemir et al, 2012[18]			/
	**Level of hypopharynx (1- vs 2-jaw)**			
	Hong et al, 2011[17]			/
	Aydemir et al, 2012[18]			/
	**Level of soft palate (MdS+MxA)**			
	Degerliyurt et al, 2008[5]		/	
	Jakobson et al, 2010[10]		/	
	**Level of tongue base (MdS)**			
	Degerliyurt et al, 2008[5]		/	
	Park et al, 2010[8]		/	
	**Level of tongue base (MdS+MxA)**			
	Degerliyurt et al, 2008[5]		/	
	Jakobson et al, 2010[10]		/	

MdS= Mandibular setback
MxA= Maxillary advancement

Table 3. Citation matrix of primary papers related to volumetric measurement of pharyngeal airway after mandibular setback surgeries

		**He *et al*, 2017[20]**	**Chris2tovam *et al,* 2015[29]**
**Primary papers**	**Total upper airway volume (MdS)**		
	Zhang R, 2011[28]	/	
	Hatab et al, 2015[21]	/	
	Park et al, 2012[26]	/	/
	Kim et al, 2010[30]	/	/
	Wang et al, 2015[31]	/	
	Uesugi et al, 2014[27]	/	/
	Hong et al, 2011[17]	/	/
	Park et al 2010[8]	/	/
	Wang et al, 2012[32]		/
	**Total upper airway volume (MdS + MdA)**		
	Hong et al, 2011[17]	/	/
	Burkhard et al, 2014[33]	/	
	Hatab et al, 2015[21]	/	
	Kim et al, 2014[34]	/	
	Park et al, 2012[26]	/	/
	Uesugi et al, 2014[27]	/	/
	Azevedo et al, 2016[22]	/	
	Zhang R, 2011[28]	/	
	Li et al, 2014[35]	/	/
	Jakobsone et al, 2010[10]	/	/
	Hart et al, 2015[23]	/	
	Gockce et al, 2014[36]	/	/
	Kim et al, 2016[24]	/	
	Hsieh et al, 2015[25]	/	
	Bruneto et al, 2014[37]		/

	Kim et al, 2013[38]		/
	Lee et al, 2009[39]		/
	Panou et al, 2013[40]		/
	**Nasopharyngeal volume (1- vs 2-jaw)**		
	Park et al, 2012[26]	/	
	Hatab et al, 2015[21]	/	
	Zhang R, 2011[28]	/	
	Uesugi et al, 2014[27]	/	
	**Oropharyngeal volume (1- vs 2-jaw)**		
	Park et al, 2012[26]	/	
	Hatab et al, 2015[21]	/	
	Zhang R, 2011[28]	/	
	Uesugi et al, 2014[27]	/	
	**Hypopharyngeal volume (1- vs 2-jaw)**		
	Park et al, 2012[26]	/	
	Hatab et al, 2015[21]	/	
	Zhang R, 2011[28]	/	
	**TOTAL volume (1- vs 2-jaw)**		
	Park et al, 2012[26]	/	/
	Hatab et al, 2015[21]	/	
	Zhang R, 2011[28]	/	
	Uesugi et al, 2014[27]	/	/
	Hong et al, 2011[17]		/

CCA= (56-43)/(43*2-43) X 100% =30.23% (very high overlap)

MdS= Mandibular setback
MxA= Maxillary advancement

Table 4. Citation matrix of primary papers related to measurements of lateral width of pharyngeal airway at base of tongue after mandibular setback surgeries

		**Mattos *et al*, 2011[2]**
Primary papers	Degerliyurt et al, 2008[5]	/
	Kawamata et al, 2000[15]	/

Table 5. Citation matrix of primary papers related to measurements of pharyngeal airway after BSSO versus VSSO surgeries

		**Al-Moraissi *et al*, 2015[3]**
Primary papers	Kitahara et al, 2010[41]	/
	Abdelrahman et al, 2011[42]	/

References:

1. Pieper D, Antoine S-L, Mathes T, Neugebauer EAM, Eikermann M. Systematic review finds overlapping reviews were not mentioned in every other overview. Journal of clinical epidemiology. 2014;67:368-75.
2. Mattos CT, Vilani GN, Sant'Anna EF, Ruellas AC, Maia LC. Effects of orthognathic surgery on oropharyngeal airway: a meta-analysis. Int J Oral Maxillofac Surg. 2011;40(12):1347-56. Epub 2011/07/26. doi: 10.1016/j.ijom.2011.06.020. PubMed PMID: 21782388.
3. Al-Moraissi EA, Al-Magaleh SM, Iskandar RA, Al-Hendi EA. Impact on the pharyngeal airway space of different orthognathic procedures for the prognathic mandible. Int J Oral Maxillofac Surg. 2015;44(9):1110-8. Epub 2015/05/31. doi: 10.1016/j.ijom.2015.05.006. PubMed PMID: 26025815.
4. Chen F, Terada K, Hua Y, Saito I. Effects of bimaxillary surgery and mandibular setback surgery on pharyngeal airway measurements in patients with Class III skeletal deformities. American journal of orthodontics and dentofacial orthopedics : official publication of the American Association of Orthodontists, its constituent societies, and the American Board of Orthodontics. 2007;131(3):372-7. Epub 2007/03/10. doi: 10.1016/j.ajodo.2005.06.028. PubMed PMID: 17346593.
5. Degerliyurt K, Ueki K, Hashiba Y, Marukawa K, Nakagawa K, Yamamoto E. A comparative CT evaluation of pharyngeal airway changes in class III patients receiving bimaxillary surgery or mandibular setback surgery. Oral surgery, oral medicine, oral pathology, oral radiology, and endodontics. 2008;105(4):495-502. Epub 2008/03/11. doi: 10.1016/j.tripleo.2007.11.012. PubMed PMID: 18329585.

6. Liukkonen M, Vahatalo K, Peltomaki T, Tiekso J, Happonen RP. Effect of mandibular setback surgery on the posterior airway size. The International journal of adult orthodontics and orthognathic surgery. 2002;17(1):41-6. Epub 2002/04/06. PubMed PMID: 11934054.
7. Muto T, Yamazaki A, Takeda S, Sato Y. Effect of bilateral sagittal split ramus osteotomy setback on the soft palate and pharyngeal airway space. Int J Oral Maxillofac Surg. 2008;37(5):419-23. Epub 2008/03/11. doi: 10.1016/j.ijom.2007.12.012. PubMed PMID: 18329854.
8. Park JW, Kim NK, Kim JW, Kim MJ, Chang YI. Volumetric, planar, and linear analyses of pharyngeal airway change on computed tomography and cephalometry after mandibular setback surgery. American journal of orthodontics and dentofacial orthopedics : official publication of the American Association of Orthodontists, its constituent societies, and the American Board of Orthodontics. 2010;138(3):292-9. Epub 2010/09/08. doi: 10.1016/j.ajodo.2009.10.036. PubMed PMID: 20816298.
9. Cakarne D, Urtane I, A. S. pharyngeal airway sagittal dimension in patients with class III skeletal dentofacial deformity before and after bimaxillary surgery Stomatologija. 2003;5:13-6.
10. Jakobsone G, Neimane L, Krumina G. Two- and three-dimensional evaluation of the upper airway after bimaxillary correction of Class III malocclusion. Oral surgery, oral medicine, oral pathology, oral radiology, and endodontics. 2010;110(2):234-42. Epub 2010/06/29. doi: 10.1016/j.tripleo.2010.03.026. PubMed PMID: 20580280.
11. Marsan G, Vasfi Kuvat S, Oztas E, Cura N, Susal Z, Emekli U. Oropharyngeal airway changes following bimaxillary surgery in Class III female adults. Journal of cranio-maxillo-facial surgery : official publication of the European Association for Cranio-Maxillo-Facial Surgery. 2009;37(2):69-73. Epub 2009/01/02. doi: 10.1016/j.jcms.2008.11.001. PubMed PMID: 19117765.
12. Mehra P, Downie M, Pita MC, Wolford LM. Pharyngeal airway space changes after counterclockwise rotation of the maxillomandibular complex. American journal of orthodontics and dentofacial orthopedics : official publication of the American Association of Orthodontists, its constituent societies, and the American Board of Orthodontics. 2001;120(2):154-9. Epub 2001/08/14. doi: 10.1067/mod.2001.114647. PubMed PMID: 11500657.
13. Chen F, Terada K, Hanada K, Saito I. Predicting the pharyngeal airway space after mandibular setback surgery. Journal of oral and maxillofacial surgery : official journal of the American Association of Oral and Maxillofacial Surgeons. 2005;63(10):1509-14. Epub 2005/09/27. doi: 10.1016/j.joms.2005.06.007. PubMed PMID: 16182920.
14. Kawakami M, Yamamoto K, Fujimoto M, Ohgi K, Inoue M, Kirita T. Changes in tongue and hyoid positions, and posterior airway space following mandibular setback surgery. Journal of cranio-maxillo-facial surgery : official publication of the European Association for Cranio-Maxillo-Facial Surgery. 2005;33(2):107-10. Epub 2005/04/05. doi: 10.1016/j.jcms.2004.10.005. PubMed PMID: 15804589.
15. Kawamata A, Fujishita M, Ariji Y, Ariji E. Three-dimensional computed tomographic evaluation of morphologic airway changes after mandibular setback osteotomy for prognathism. Oral surgery, oral medicine, oral pathology, oral radiology, and endodontics. 2000;89(3):278-87. Epub 2000/03/10. PubMed PMID: 10710450.

16. Hwang S, Chung CJ, Choi YJ, Huh JK, Kim KH. Changes of hyoid, tongue and pharyngeal airway after mandibular setback surgery by intraoral vertical ramus osteotomy. The Angle orthodontist. 2010;80(2):302-8. Epub 2009/11/13. doi: 10.2319/040209-188.1. PubMed PMID: 19905855.
17. Hong JS, Park YH, Kim YJ, Hong SM, Oh KM. Three-dimensional changes in pharyngeal airway in skeletal class III patients undergoing orthognathic surgery. Journal of oral and maxillofacial surgery : official journal of the American Association of Oral and Maxillofacial Surgeons. 2011;69(11):e401-8. Epub 2011/05/17. doi: 10.1016/j.joms.2011.02.011. PubMed PMID: 21571419.
18. Aydemir H, Memikoglu U, Karasu H. Pharyngeal airway space, hyoid bone position and head posture after orthognathic surgery in Class III patients. The Angle orthodontist. 2012;82(6):993-1000. Epub 2012/04/17. doi: 10.2319/091911-597.1. PubMed PMID: 22500578.
19. Kobayashi T, Funayama A, Hasebe D, Kato Y, Yoshizawa M, Saito C. Changes in overnight arterial oxygen saturation after mandibular setback. The British journal of oral & maxillofacial surgery. 2013;51(4):312-8. Epub 2012/08/03. doi: 10.1016/j.bjoms.2012.07.004. PubMed PMID: 22853977.
20. He J, Wang Y, Hu H, Liao Q, Zhang W, Xiang X, et al. Impact on the upper airway space of different types of orthognathic surgery for the correction of skeletal class III malocclusion: A systematic review and meta-analysis. International journal of surgery (London, England). 2017;38:31-40. Epub 2016/12/29. doi: 10.1016/j.ijsu.2016.12.033. PubMed PMID: 28027997.
21. Hatab NA, Konstantinovic VS, Mudrak JK. Pharyngeal airway changes after mono- and bimaxillary surgery in skeletal class III patients: Cone-beam computed tomography evaluation. Journal of cranio-maxillo-facial surgery : official publication of the European Association for Cranio-Maxillo-Facial Surgery. 2015;43(4):491-6. Epub 2015/03/22. doi: 10.1016/j.jcms.2015.02.007. PubMed PMID: 25794643.
22. Azevedo MS, Machado AW, Barbosa Ida S, Esteves LS, Rocha VA, Bittencourt MA. Evaluation of upper airways after bimaxillary orthognathic surgery in patients with skeletal Class III pattern using cone-beam computed tomography. Dental press journal of orthodontics. 2016;21(1):34-41. Epub 2016/03/24. doi: 10.1590/2177-6709.21.1.034-041.oar. PubMed PMID: 27007759; PubMed Central PMCID: PMCPMC4816583.
23. Hart PS, McIntyre BP, Kadioglu O, Currier GF, Sullivan SM, Li J, et al. Postsurgical volumetric airway changes in 2-jaw orthognathic surgery patients. American journal of orthodontics and dentofacial orthopedics : official publication of the American Association of Orthodontists, its constituent societies, and the American Board of Orthodontics. 2015;147(5):536-46. Epub 2015/04/29. doi: 10.1016/j.ajodo.2014.12.023. PubMed PMID: 25919099.
24. Kim HS, Kim GT, Kim S, Lee JW, Kim EC, Kwon YD. Three-dimensional evaluation of the pharyngeal airway using cone-beam computed tomography following bimaxillary orthognathic surgery in skeletal class III patients. Clinical oral investigations. 2016;20(5):915-22. Epub 2015/09/04. doi: 10.1007/s00784-015-1575-4. PubMed PMID: 26330061.

25. Hsieh YJ, Chen YC, Chen YA, Liao YF, Chen YR. Effect of bimaxillary rotational setback surgery on upper airway structure in skeletal class III deformities. Plastic and reconstructive surgery. 2015;135(2):361e-9e. Epub 2015/01/30. doi: 10.1097/prs.0000000000000913. PubMed PMID: 25626820.
26. Park SB, Kim YI, Son WS, Hwang DS, Cho BH. Cone-beam computed tomography evaluation of short- and long-term airway change and stability after orthognathic surgery in patients with Class III skeletal deformities: bimaxillary surgery and mandibular setback surgery. Int J Oral Maxillofac Surg. 2012;41(1):87-93. Epub 2011/10/26. doi: 10.1016/j.ijom.2011.09.008. PubMed PMID: 22024138.
27. Uesugi T, Kobayashi T, Hasebe D, Tanaka R, Ike M, Saito C. Effects of orthognathic surgery on pharyngeal airway and respiratory function during sleep in patients with mandibular prognathism. Int J Oral Maxillofac Surg. 2014;43(9):1082-90. Epub 2014/07/17. doi: 10.1016/j.ijom.2014.06.010. PubMed PMID: 25027545.
28. Zhang R. Dimensioanl morphological changes of pharyngeal airway after orthognathic surgery for patients with skeletal class III dentomaxillofacial deformity and its comparative study: Shanghai Jiatong University; 2011.
29. Christovam IO, Lisboa CO, Ferreira D, Cury-Saramago AA, Mattos CT. Upper airway dimensions in patients undergoing orthognathic surgery: a systematic review and meta-analysis. International Journal of Oral and Maxillofacial Surgery. 2016;45(4):460-71. doi: 10.1016/j.ijom.2015.10.018. PubMed PMID: WOS:000372938300008.
30. Kim NR, Kim YI, Park SB, DS H. Three dimensional cone-beam CT study of upper airway change after mandibular setback surgery for skeletal class III malocclusion patients. Korean J Orthod. 2010;40:145-55.
31. Wang H, Qi S, Yan M, Zhang C, Ren S, Zhang J. [Cone-beam computed tomography evaluation of upper airway change in skeletal Class III patients after orthodontic-mandibular setback surgery]. Zhonghua kou qiang yi xue za zhi = Zhonghua kouqiang yixue zazhi = Chinese journal of stomatology. 2015;50(10):615-8. Epub 2016/01/14. PubMed PMID: 26757631.
32. Wang HW, Wang JG, Qi SQ, Cai ZF, Li XH. [Three-dimensional analysis of pharyngeal airway in skeletal Class III patients after sagittal split ramus osteotomy]. Zhonghua kou qiang yi xue za zhi = Zhonghua kouqiang yixue zazhi = Chinese journal of stomatology. 2012;47(4):221-4. Epub 2012/07/18. doi: 10.3760/cma.j.issn.1002-0098.2012.04.007. PubMed PMID: 22800700.
33. Burkhard JP, Dietrich AD, Jacobsen C, Roos M, Lubbers HT, Obwegeser JA. Cephalometric and three-dimensional assessment of the posterior airway space and imaging software reliability analysis before and after orthognathic surgery. Journal of cranio-maxillo-facial surgery : official publication of the European Association for Cranio-Maxillo-Facial Surgery. 2014;42(7):1428-36. Epub 2014/05/28. doi: 10.1016/j.jcms.2014.04.005. PubMed PMID: 24864074.
34. Kim MA, Kim BR, Youn JK, Kim YJ, Park YH. Head posture and pharyngeal airway volume changes after bimaxillary surgery for mandibular prognathism. Journal of cranio-maxillo-facial surgery : official publication of the European Association for Cranio-Maxillo-Facial Surgery. 2014;42(5):531-5. Epub 2013/10/02. doi: 10.1016/j.jcms.2013.07.022. PubMed PMID: 24080139.

35. Li YM, Liu JL, Zhao JL, Dai J, Wang L, Chen JW. Morphological changes in the pharyngeal airway of female skeletal class III patients following bimaxillary surgery: a cone beam computed tomography evaluation. Int J Oral Maxillofac Surg. 2014;43(7):862-7. Epub 2014/04/05. doi: 10.1016/j.ijom.2014.03.009. PubMed PMID: 24697918.
36. Gokce SM, Gorgulu S, Gokce HS, Bengi AO, Karacayli U, Ors F. Evaluation of pharyngeal airway space changes after bimaxillary orthognathic surgery with a 3-dimensional simulation and modeling program. American journal of orthodontics and dentofacial orthopedics : official publication of the American Association of Orthodontists, its constituent societies, and the American Board of Orthodontics. 2014;146(4):477-92. Epub 2014/09/30. doi: 10.1016/j.ajodo.2014.06.017. PubMed PMID: 25263151.
37. Brunetto DP, Velasco L, Koerich L, Araujo MT. Prediction of 3-dimensional pharyngeal airway changes after orthognathic surgery: a preliminary study. American journal of orthodontics and dentofacial orthopedics : official publication of the American Association of Orthodontists, its constituent societies, and the American Board of Orthodontics. 2014;146(3):299-309. Epub 2014/08/31. doi: 10.1016/j.ajodo.2014.05.024. PubMed PMID: 25172252.
38. Kim MA, Kim BR, Choi JY, Youn JK, Kim YJ, Park YH. Three-dimensional changes of the hyoid bone and airway volumes related to its relationship with horizontal anatomic planes after bimaxillary surgery in skeletal Class III patients. The Angle orthodontist. 2013;83(4):623-9. Epub 2013/01/15. doi: 10.2319/083112-700.1. PubMed PMID: 23311605.
39. Lee YS, Baik HS, Lee KJ, HS Y. The structural change in the hyoid bone and upper airway after orthognathic surgery for skeletal class III anterior open bite patients using 3-dimensional computed tomography. Korean J Orthod. 2009;39:72-82.
40. Panou E, Motro M, Ates M, Acar A, Erverdi N. Dimensional changes of maxillary sinuses and pharyngeal airway in Class III patients undergoing bimaxillary orthognathic surgery. The Angle orthodontist. 2013;83(5):824-31. Epub 2013/02/27. doi: 10.2319/100212-777.1. PubMed PMID: 23438197.
41. Kitahara T, Hoshino Y, Maruyama K, In E, Takahashi I. Changes in the pharyngeal airway space and hyoid bone position after mandibular setback surgery for skeletal Class III jaw deformity in Japanese women. American journal of orthodontics and dentofacial orthopedics : official publication of the American Association of Orthodontists, its constituent societies, and the American Board of Orthodontics. 2010;138(6):708.e1-10; discussion -9. Epub 2010/12/07. doi: 10.1016/j.ajodo.2010.06.014. PubMed PMID: 21130322.
42. Abdelrahman TE, Takahashi K, Tamura K, Nakao K, Hassanein KM, Alsuity A, et al. Impact of different surgery modalities to correct class III jaw deformities on the pharyngeal airway space. The Journal of craniofacial surgery. 2011;22(5):1598-601. Epub 2011/10/01. doi: 10.1097/SCS.0b013e31822e5fc2. PubMed PMID: 21959395.
